# Monitoring mammary gland development in preweaning dairy heifers using ultrasound imaging

**DOI:** 10.3168/jdsc.2024-0586

**Published:** 2024-05-31

**Authors:** Alysia L. Vang, Tiago Bresolin, Waneska S. Frizzarini, Joana P. Campolina, Guilherme L. Menezes, Guilherme J.M. Rosa, Joao R.R. Dorea, Laura L. Hernandez

**Affiliations:** 1 Department of Animal and Dairy Sciences, University of Wisconsin–Madison, Madison, WI 53706; 2 Department of Animal Sciences, University of Illinois Urbana-Champaign, Urbana, IL 61801

## Abstract

Existing literature on the long-term effects of nutritional manipulation on milk production is incomplete and inconsistent as animals are typically culled before lactation. In addition, longitudinal studies are rarely conducted due to high costs and constraints related to tissue sampling; therefore, additional research is necessary to better understand the effect of early nutrition and growth on subsequent growth and milk production. Thirty Holstein heifer calves were pair-fed high (H; 27% CP and 20% fat) or low (L; 22% CP and 15%) milk replacers. Mammary gland ultrasound images and BW were collected twice weekly, and blood samples weekly, for the first 8 wk of life. Calves fed H had higher ADG and mammary gland growth through 8 wk of life. Calves from the H group also had larger fat pads at 8 wk of age. Glucose and insulin concentrations were increased in H calves compared with L calves, whereas nonesterified fatty acid concentrations were not different between treatments. Our results suggest that ultrasound is a practical, noninvasive tool to monitor udder development preweaning, although additional research is necessary to improve image analysis techniques to analyze tissue composition. Our preliminary work provides data suggesting that the use of mammary gland ultrasound could provide a method of longitudinal analysis of the effects of preweaning nutrition on milk production.

## Highlights

• Growth differences were detected using ultrasound imaging.
• Animals given milk replacer higher in protein and fat content displayed increased parenchymal and mammary fat pad growth from 3 weeks through 8 weeks of life.
• Mammary gland ultrasound imaging is a powerful tool to monitor mammary parenchyma growth in young animals.

## Graphical Abstract

**Figure:**
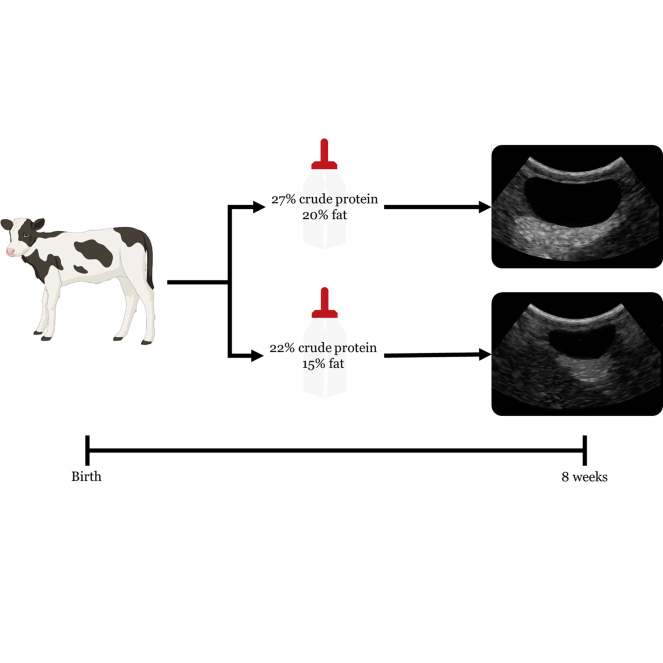
**Summary:** Calves were fed 2 different milk replacers (high and low) to create mammary gland growth differences that could be detected by ultrasound imaging. The animals fed the high milk replacer displayed increased growth from 3 weeks through 8 weeks, measured as area by ultrasound imaging. Ultrasound was successful in capturing growth of the mammary gland preweaning and has the potential to become a powerful tool to monitor the development of parenchymal tissue in preweaning dairy calves as differences in growth through measurement of parenchymal area were detected. Created with BioRender.com.

Numerous studies have postulated that nutrition plays a pivotal role in mammary gland development of dairy cows from birth through parturition, although results are mixed as to the effect on milk production (Shamay et al., 2005; Raeth-Knight et al., 2009; Terré et al., 2009; Davis Rincker et al., 2011). Because raising replacement heifers is one of the largest expenses on a dairy farm, it is worthwhile to explore opportunities to better select for profitable heifers (Zwald et al., 2007). The current selection strategy of utilizing genetic merit does not account for udder development because phenotype is influenced by genotype as well as environmental factors; therefore, the addition of a method to monitor udder development would be relevant and provide additional selection criteria (Stevenson and Ahmadzadeh, 2011). Although research has been conducted to monitor and measure mammary gland development in young heifers, data are incomplete and inconsistent as milk production data are typically missing because calves and heifers are culled for tissue collection and methods of quantifying growth and development vary between studies. Ultrasound technology is widely used in visualization and diagnosis in veterinary medicine (Braun, 2005; Quintela et al., 2012). Researchers have also explored its use for tracking udder development, although additional research is necessary to validate ultrasound as an appropriate and sensitive method for determining productive capacity of the tissue (Nishimura et al., 2011).

The objective of this preliminary study was to evaluate ultrasound as a method of monitoring mammary gland development in calves. We hypothesized that ultrasound could be used to detect differences in growth by feeding 2 different milk replacers for the first 7 wk of life. All procedures were approved by the Animal Care and Use Committee of the University of Wisconsin–Madison (A006270-R01). Thirty Holstein heifer calves (37.2 ± 1.11 kg) were pair-fed high (**H**; n = 15) or low (**L**; n = 15) milk replacers for 7 wk at the University of Wisconsin–Madison's Blaine Dairy Cattle Center in Arlington, Wisconsin. Following a 5-d adaption period, the L calves received 2 quarts (1.9 L) of low protein and fat milk replacer (Herd Maker Protein Blend, Land O'Lakes; 22% CP, 15% fat), whereas the H calves received 1 gallon of high protein and fat milk replacer twice daily (7.6 L; Cow's Match ColdFront Protein Blend, Land O' Lakes; 27% CP, 20% fat). Starter (18% CP guaranteed analysis; UW Calf Starter–Medicated Rum/Clar, Vita Plus, Lake Mills Feed and Grain Inc., Lake Mills, WI) was available beginning at 7 d and gradual weaning began on d 42, with calves fully weaned from milk replacer by 49 d of age. The starter amount provided to the L calves was adjusted daily according to the amounts the paired H calves consumed the previous day. At 8 wk of age, the calves transitioned to ad libitum grower grain (15% CP guaranteed analysis; Vita Plus, Lake Mills Feed and Grain Inc., Lake Mills, WI). At 12 wk of age, the heifers moved to the Marshfield Agricultural Research Station in Stratford, Wisconsin, where they were transitioned from grower grain to TMR and fed standard herd diets through the first lactation.

To conduct mammary ultrasound, the calves were placed in the lying position for the procedure. Body weight and ultrasound images were collected twice weekly for every calf (Mindray Z5 Ultrasound, Mindray 65C15EA MHz Micro-Convex Ultrasound Transducer), and blood samples were collected once a week for 8 wk. The 1,414 ultrasound images were manually segmented, and the parenchymal (**PAR**) area was extracted. Image segmentation involves partitioning of pixels into discrete groups, in this case, parenchyma. The images were analyzed using Matlab 2021b (MathWorks) and statistical analysis was conducted using a linear mixed model in R version 4.0.4 (https://www.r-project.org/). Linear mixed models were used to analyze PAR area and included the fixed effects of treatment (H or L), quarter, and the interaction of treatment and quarter, as well as the random effect of animal. Images at 8 wk of age were also annotated using QuPath Version 0.4.3 and mean echogenicity was measured by calculation of mean pixel brightness (Bankhead et al., 2017). Linear fixed models were used to analyze PAR and mammary fat pad (**MFP**) mean echogenicity at 8 wk including the effects of diet (L or H) and birth weight (**BRW**). Blood samples were analyzed for glucose using the FujiFilm Glucose Autokit (FujiFilm Wako Pure Chemical Corporation, Chuo-Ku, Osaka, Japan). The intra- and interassay coefficients of variation for glucose were 8.66% and 11.3%, respectively. Insulin was analyzed using the Mercodia Bovine Insulin ELISA kit (Mercodia, Uppsala, Sweden). The intra- and interassay CV for insulin were 7.21% and 7.32%, respectively. A Chemwell analyzer (Awareness Technology Inc.) was used to measure nonesterified fatty acid (**NEFA**) concentrations (C514–0A; CataChem NEFA, Oxford, CT). The intra- and interassay CV for NEFA were 4.14% and 4.38%, respectively. Linear mixed models with repeated measures were used to analyze glucose, insulin, and NEFA content in blood plasma including the fixed effects of treatment (L or H), week (1–8), and the interaction of treatment × week, the covariate of BRW, and the random effect of calf.

Glucose, insulin, and NEFA concentrations were measured in weekly plasma samples collected. Glucose concentrations were elevated in H calves compared with L calves (*P* = 0.02; Table 1). Insulin concentrations were also increased in H calves compared with L calves (*P* = 0.01; Table 1). Calf BRW and week of age were positively associated with increasing insulin concentrations (*P* = 0.008, *P* = 0.004; Table 1). Although NEFA concentrations were not significantly different among the 2 treatments, they did increase with age (*P* = 0.39, *P* < 0.001; Table 1). Our results suggest that increasing protein and fat content in milk replacer increases glucose and insulin levels in calves, which is consistent with previous research which states that higher concentrations of glucose and insulin indicate superior metabolic characteristics and displayed superior growth, although the long-term metabolic and growth consequences are unclear as measurements were not made after the treatment period in this study and should be explored in the future (Ockenden et al., 2023).

**Table 1:** Measurements from birth through 8 wk^1^

Variable	No.	Treatment		*P*-value			
		L	H	D	W	D × W	BRW
Plasma measurement							
NEFA (mmol/L)	30	0.21 ± 0.021	0.22 ± 0.013	0.39	<0.001	0.70	0.09
Glucose (mmol/L)	30	4.33 ± 0.16	4.77 ± 0.15	0.02	0.13	0.97	0.68
Insulin (mU/L)	30	168 ± 1.06	197 ± 1.51	0.01	0.004	0.78	0.008
	No.	L	H	D		BRW	
Parenchymal measurement from 1 to 8 wk							
PAR area (mm^2^)							
Week 1	30	21.9 ± 1.02	21.3 ± 0.97	0.98		0.07	
Week 2	30	19.0 ± 0.90	20.0 ± 0.89	0.20		0.006	
Week 3	30	17.5 ± 0.65	21.5 ± 0.78	<0.001		0.016	
Week 4	30	20.3 ± 0.83	24.3 ± 1.27	0.010		0.57	
Week 5	30	19.5 ± 1.03	25.5 ± 1.10	<0.001		0.006	
Week 6	30	24.7 ± 1.31	46.0 ± 3.07	<0.001		0.85	
Week 7	30	38.1 ± 2.64	53.8 ± 4.2	0.007		0.23	
Week 8	30	56.5 ± 3.87	80.3 ± 5.87	0.002		0.49	
MFP area wk 8	28	56.9 ± 5.30	86.9 ± 7.00	0.02		0.15	
PAR mean echogenicity wk 8	30	0.086 ± 0.011	0.083 ± 0.007	0.50		0.55	
MFP mean echogenicity wk 8	30	0.36 ± 0.012	0.37 ± 0.014	0.19		0.57	

1 Mean (± SE) values of birth weight (BRW), nonesterified fatty acids (NEFA), glucose, insulin, parenchymal (PAR) area and echogenicity, and mammary fat pad (MFP) area and mean echogenicity at wk 8 by diet, high (H) and low (L), with *P*-values indicating significant associations with diet (D), week (W), interaction of diet and week (D × W), and BRW on the response variables.

Total PAR area increased over time in both treatments as depicted in Figure 1, Figure 2. Calves fed the H diet had increased PAR area beginning at 3 wk of age (Table 1, Figure 1). Our results are consistent with previous findings in which PAR size and weight increased with enhanced milk replacer feedings (i.e., increased protein and fat content), although previous papers required culling of animals for tissue collection (Brown et al., 2005; Geiger et al., 2016). Dramatic growth occurred at 5 wk of age in both treatment groups, although the H calves displayed increased growth compared with the L calves. This may be due to compounding effects of nutrition and increased hormone sensitivity due to advanced maturation in the H calves, as one study found that calves fed an enhanced diet had increased PAR and MFP growth in response to exogenous estrogen treatment immediately postweaning (Geiger et al., 2016). High nutritional diets have been shown to accelerate maturation of the hypothalamic-pituitary-ovarian axis as it was demonstrated that calves fed high nutritional diets from 3 to 21 wk of life expressed increased hypothalamic-pituitary-ovarian axis sensitivity to estradiol feedback when subjected to a GnRH challenge at 19 wk of age, compared with animals fed a moderate diet whose synthesis and secretion of gonadotropins were characteristic of postnatal calf patterns independent of gonadal steroids (Kelly et al., 2020). In tandem with advanced maturity, animals fed the high nutritional diets also displayed increased allometric growth, with increased metabolic organ, total reproductive tract, uterine, and ovarian tissue weight compared with their moderate counterparts. In another study calves were ovariectomized between 1 and 3 mo of age and euthanized at 6 mo of age to investigate the effect of ovariectomy on estrogen receptor expression and mammary epithelial cell proliferation in the mammary gland (Berry et al., 2003). Calves ovariectomized before 6 wk of age displayed the least amount of epithelial tissue compared with the control and animals ovariectomized after 6 wk of age as development was minimal after the procedure. Ovariectomy hindered mammary development in animals ovariectomized after 6 wk of age, although the calves displayed increased development compared with the calves that underwent the procedure before 6 wk of age. Termination of mammary gland development in calves ovariectomized before 6 wk of age suggests that the first 6 wk of life is a critical period in development, although further research is required to characterize hormonal profiles of young heifers and the relationship between early nutrition, hormones, and development.

**Figure 1:**
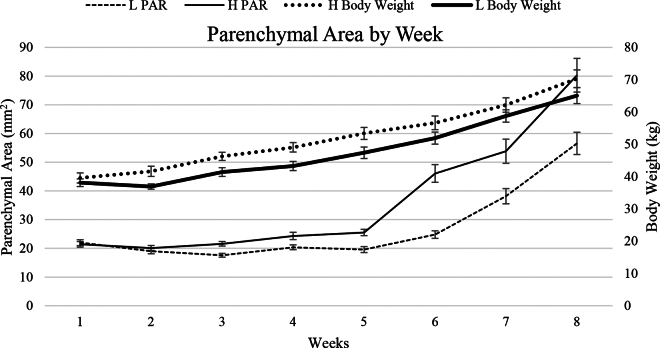
Parenchymal area was similar between the L and H treatments during wk 1 and 2, although beginning at 3 wk of age, H animals began displaying larger parenchymal areas and continued to do so through 8 wk of age. Average BW was similar between treatments during wk 1 and became significantly (*P* < 0.05) different between 2 and 6 wk. During wk 7 and 8 average BW were similar between treatments due to weaning.

**Figure 2:**
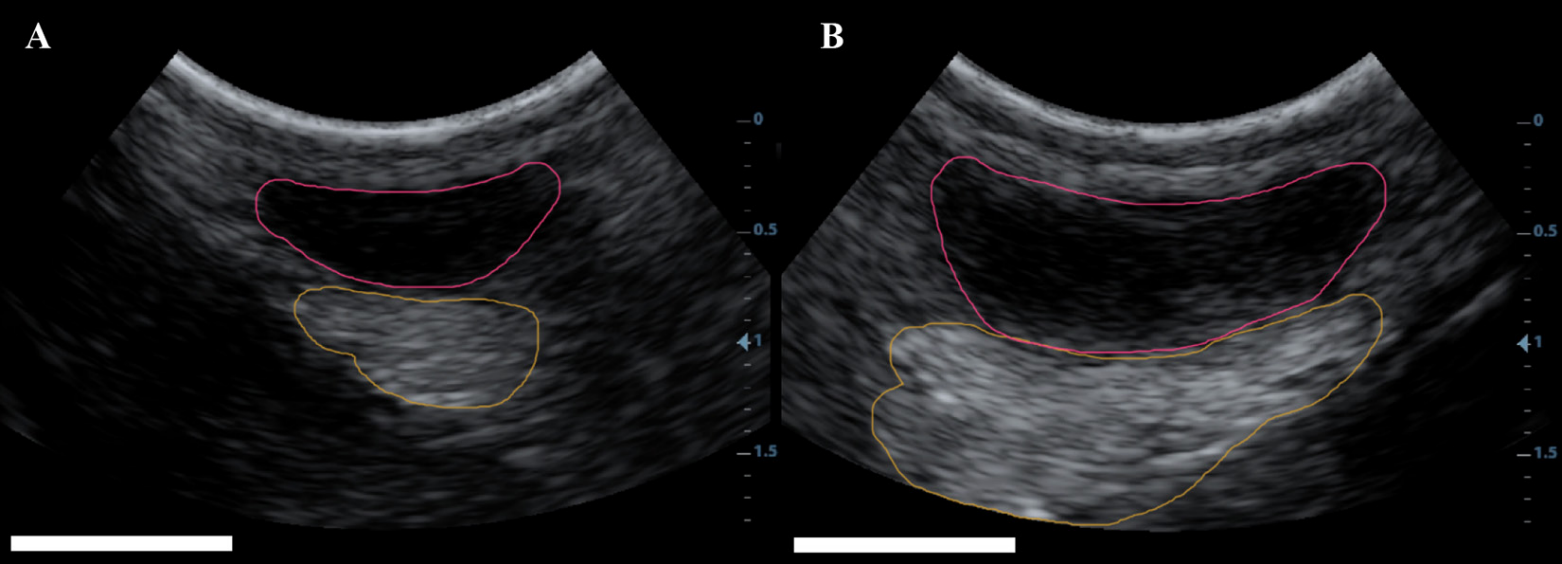
Annotated image of the rear left quarters of paired 8-wk-old calves on the L treatment (A) and H treatment (B). The parenchyma is outlined in pink and the mammary fat pad is outlined in yellow. The white bar measures 1 cm.

The PAR and MFP echogenicity did not significantly differ between dietary treatment groups (Table 1). There is limited research analyzing mean echogenicity of developing mammary glands, although another study classified pixels based on pixel value and demonstrated accurate quantification of secretory tissue in periparturient heifers (Strzetelski et al., 2004). This method may be applicable to calves at weaning as PAR grows into the fat pad; therefore, additional research is required to quantify ductal structures noninvasively in calves. While use of the ultrasound imaging of the mammary gland allowed collection of PAR development during the first 8 wk of age, additional research with larger sample sizes is warranted to further improve the use of ultrasound to assess mammary gland development over time. Specific efforts should be made to compare PAR and MFP over time and how nutritional manipulations at different stages of life to determine how these measurements are associated with milk production. Additional research is also required to validate the impact on mammary gland microstructure, both during development and lactation, and their relationships to the production of milk components.

In conclusion, ultrasound imaging was successfully used to analyze PAR area from birth through 8 wk of age. Calves fed a milk replacer enhanced in protein and fat content for the first 7 wk of life had larger PAR areas beginning at 3 wk of age and maintained increased PAR area through 8 wk. The MFP area was also larger in H animals at 8 wk of age. Mean echogenicity of PAR and the MFP were not different between treatment groups. Methods of analyzing PAR echogenicity of ultrasound images should be further explored as mean echogenicity may not capture tissue development and microstructure precisely in calves. As mentioned previously, researchers have showed that classification of individual pixels based on pixel value produced adequate proportions of secretory tissue in periparturient heifers (Strzetelski et al., 2004). It is possible that before gestation and lactation, a similar method in which individual pixels are analyzed rather than mean pixel value of the entire imaged gland may be a better representation of tissue composition. Further development of noninvasive approaches for quantifying mammary gland development are necessary to allow for longitudinal analysis of development. Our preliminary data suggest that features extracted from ultrasound mammary gland images can be a powerful tool to monitor the development of PAR tissue in preweaning dairy calves as differences in growth through measurement of PAR area were detected.
